# Computation of topographic and three-dimensional atomic force microscopy images of biopolymers by calculating forces

**DOI:** 10.1007/s12551-023-01167-1

**Published:** 2023-11-27

**Authors:** Takashi Sumikama

**Affiliations:** 1 PRESTO, JST, Kawaguchi, Saitama 332-0012 Japan; 2 Nano Life Science Institute (WPI-NanoLSI), Kanazawa University, Kanazawa, 920-1192 Japan

**Keywords:** AFM, Simulation, Biomolecule, Free energy, Jarzynski, Nonequilibrium

## Abstract

Atomic force microscopy (AFM) is widely utilized to visualize the molecular motions of biomolecules. Comparison of experimentally measured AFM images with simulated AFM images based on known structures of biomolecules is often necessary to elucidate what is actually resolved in the images. Experimental AFM images are generated by force measurements; however, conventional AFM simulation has been based on geometrical considerations rather than calculating forces using molecular dynamics simulations due to limited computation time. This letter summarizes recently developed methods to simulate topographic and three-dimensional AFM (3D-AFM) images of biopolymers such as chromosomes and cytoskeleton fibers. Scanning such biomolecules in AFM measurements usually results in nonequilibrium-type work being performed. As such, the Jarzynski equality was employed to relate the nonequilibrium work to the free energy profiles, and the forces were calculated by differentiating the free energy profiles. The biomolecules and probes were approximated using a supra-coarse-grained model, allowing the simulation of force-distance curves in feasible time. It was found that there is an optimum scanning velocity and that some of polymer structures are resolved in the simulated 3D-AFM images. The theoretical background adopted to rationalize the use of small probe radius in the conventional AFM simulation of biomolecules is clarified.

## Introduction

Atomic force microscopy (AFM) is a type of scanning probe microscopes and measures forces between the probe and the samples (Morita et al. 2002). AFM can visualize topographic images of samples by drawing surfaces on which a constant force, called the setpoint, acts. That is, AFM images show isoforce surfaces on the samples. Strictly speaking, it is iso-frequency shift images in frequency modulation AFM and iso-amplitude shift images in amplitude modulation AFM, but here “isoforce” is used for simplicity.

AFM was originally developed for application to samples in a vacuum (Binnig et al. 1986), and later AFM measurement at the atomic resolution became possible even in liquid as well (Fukuma et al. 2005). It is now applied to biomolecules (Müller et al. 1999), and even dynamic molecular motions are visualized after the development of high-speed AFM (Ando 2022). Another state-of-the-art AFM is the three-dimensional AFM (3D-AFM) (Fukuma and Garcia 2018). It maps forces between the probe and the samples in 3D space to visualize 3D distribution of molecules by measuring force-distance curves, also known as force spectroscopy, at each *x* and *y* position, where *z* is the height from the substrate surface. This technology was first applied to solvent molecules on substrate surfaces (Fukuma et al. 2010) and recently was used to visualize 3D organization of intracellular components in living biological cells (Penedo et al. 2021).

It should be noted that “topography” in AFM is an isoforce surface. The molecular shapes seen in the topography generally reflect the forces, but they are not in perfect agreement. Therefore, it is important to calculate the forces to simulate AFM images. Such simulations in a vacuum have a rich history and refer to the papers by Hofer et al. (2003) and Jelínek (2017) for such simulations. This letter concentrates on the simulations of AFM images for biomolecules.

Conventional simulations of AFM images of biomolecules were based on a geometrical consideration by assuming hard collisions between the probe and the samples (Markiewicz and Goh 1995; Nečas and Klapetek 2012; Niina et al. 2020; Amyot and Flechsig 2020). Typically, the probe is considered to be a cone shape, and the geometry of the tip is assumed to be characterized by two parameters: cone angle and tip radius. Using these parameters, simulated AFM images are computed as surfaces over which the probe moves while keeping a fixed distance from the sample. Accordingly, simulated AFM images in biological systems are generally equidistance surfaces from the samples, as if they were drawn using a pair of compasses. However, as is well known, biological molecules are soft and move in liquid. For example, it was found that structural parts that change their configurations in tens of nanosecond are difficult to be resolved with AFM measurements (Sumino et al. 2013). As such, it is beneficial to simulate AFM images of biomolecules by calculating forces.

## Computations of force-distance curves at solid–liquid interfaces

Apart from biomolecules, it is important to see how force-distance curves were computed in solid–liquid systems. Two papers reported the calculations of force-distance curves on the CaF_2_ and calcite substrates in water by assuming that the probe is also made by CaF_2_ or calcite (Watkins and Shulger 2010; Reischl et al. 2013). Their strategy was to (1) compute the free energy profiles or potential of mean forces (PMF) and (2) differentiate them with respect to height to yield the force-distance curves. To compute the free energy profiles, free energy calculation methods such as the umbrella sampling or the free energy perturbation method can be used (Frenkel and Smit 2002).

Another prediction is based on a theoretical approach (Watkins and Reischl 2013; Amano et al. 2013). Their strategy is to (1) compute water distribution on the solid substrates and (2) convert water distribution to force-distance curves via a theoretical formula. To compute the water distribution, we can use MD simulation (Watkins and Reischl 2013) or integral equation theory (Amano et al. 2013). To establish a theoretical formula to convert from distribution to force-distance curve, the tip was assumed to be the same as solvent (i.e., water molecules); thus, this approach is called as solvent tip approximation (STA). This is an approximation, but computational cost is much cheaper than computing the free energy profiles.

**Fig. 1:**
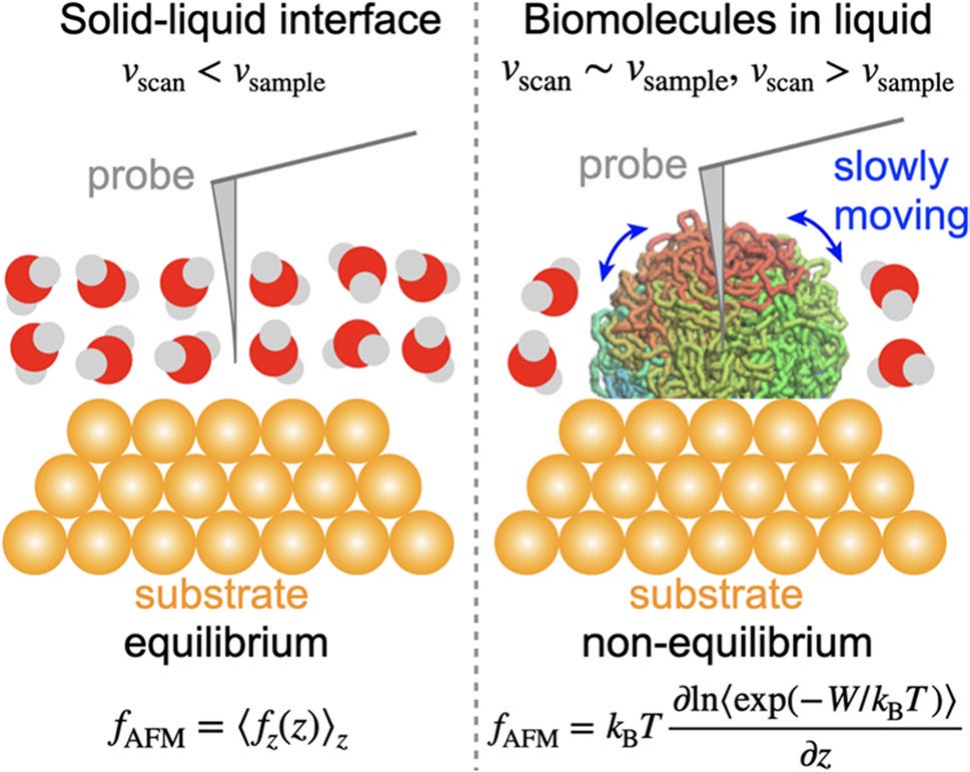
Schematic pictures of scanning in solid–liquid interface and biomolecules in liquid. The biopolymer is a single fiber with colored from red (one end) to blue (the other end)

One important thing is that both approaches assume that the systems are in equilibrium. Note that all these studies drew the force-distance curves in solid-water interfaces. Typical tip vertical scanning velocity (*v*_scan_) ranges from 1 to 100 µm/s. Accordingly, when *v*_scan_ = 100 µm/s, tip moves only 1 Å in 1 µs. Meanwhile, in 1 µs, water molecules are considered to take almost all orientations and directions around the tip, since the orientational lifetime of water molecules is in the order of picosecond (Ohmine and Tanaka 1993). This short lifetime of water molecule orientational motion rationalizes the assumption where the systems are in equilibrium. Generally, when the velocity of samples (*v*_sample_) are faster than *v*_scan_, the systems reach equilibrium during an oscillation cycle (Fig. 1, left).

Using thermodynamic integration, the Helmholtz free energy or PMF difference between state *i* and *j* (Δ*F*_*ij*_) is calculated by the following equation (Frenkel and Smit 2002):

$${\Delta F}_{ij}={\int }_{{\lambda }_{i}}^{{\lambda }_{j}}{{\big \langle} \frac{\partial V(\lambda )}{\partial \lambda }{\big \rangle} }_{\lambda }d\lambda ,$$

where *V* and *λ* are the potential energy function and a coupling parameter, respectively. The angular bracket denotes an ensemble average. To calculate the force-distance curve, *λ* is the height from the substrate surface, *z*. It is natural to assume that the initial state *i* is the moment at the surface (*z* = 0), so *λ*_*i*_ is 0 and *λ*_*j*_ is *z*. Accordingly, the above equation becomes

$$F\left(z\right)={\int }_{0}^{z}{{\big \langle} \frac{\partial V\left(z\right)}{\partial z}{\big \rangle} }_{z}dz= -{\int }_{0}^{z}{\langle {f}_{z}\left(z\right)\rangle }_{z}dz,$$

where the lowercase *f* stands for the force, and *f*_*z*_ is the *z*-component of *f*. As noted above, the forces in AFM measurements (*f*_AFM_) are the derivative of PMF:

1 $${f}_{\mathrm{AFM}}=-\frac{\partial F\left(z\right)}{\partial z}={\langle {f}_{z}(z)\rangle }_{z}$$

Accordingly, in this case (*v*_scan_ < *v*_sample_), the forces in AFM measurement are equivalent to the so-called mean force (Fig. 1, left).

## Difficulties in computing force-distance curves for biomolecules from all-atom MD simulations

It is worthwhile to estimate the simulation time for computing a force-distance curve using all-atom MD simulations. Force-distance curves in AFM measurements usually have peaks up to the Debye distance, which depends on the ion concentration and approximately 1 nm from the sample at 100 mM. Considering the molecular motion of biomolecules, the distance to be computed is 2 nm at the shortest. When *v*_scan_ = 100 µm/s, the simulation time is 20 µs. Depending on the machine configuration, the simulation speed using a GPU and the AMBER package is currently ~ 200 ns/day for a system having approximately 100,000 atoms, which is an average or relatively small system for biological simulations. Thus, it takes 100 days to compute a single force-distance curve for this system and more days for larger systems. In particular, 10,000 force-distance curves have to be computed to simulate a 3D-AFM image, when the 3D-AFM image is composed of 100 × 100 curves. Accordingly, coarse-grained approaches should be taken (Arkhipov et al. 2009).

Another problem is that most biomolecules move (relative to solvent) very slowly. For example, the gates of ion channels move in hundreds of microseconds (Jensen et al. 2012), and dynamics of chromosome takes milliseconds or longer (Nozaki et al. 2017). Thus, most biological systems never reach equilibrium at typical scan velocity (Fig. 1, right). As such, the above approach to calculate PMF cannot be employed, and another approach should be developed to simulate force-distance curves.

## A method to compute 3D-AFM images of biopolymers

Sumikama et al. developed a method to compute 3D-AFM images of biopolymers (Sumikama et al. 2022). DNA and proteins, two of the major class of biomolecules, are biopolymers. Chromosomes and probes were approximated by bead-spring polymer model, a kind of supra-coarse-grained simulation model. The simulation system consisted of a fractal globule polymer, previously considered as a model for the interphase chromosomes (Lieberman-Aiden et al. 2009), and the probe.

To calculate the force-distance curves inside the globular polymer, the Jarzynski equality was employed (Jarzynski 1997). From this equality, we can estimate the free energy difference by the work done in the nonequilibrium process (*W*):

2 $$\Delta F=-{k}_{\mathrm{B}}T\mathrm{ln}\langle \mathrm{exp}(-W/{k}_{\mathrm{B}}T)\rangle$$

where *k*_B_ and *T* are the Boltzmann constant and the temperature, respectively. The forces in AFM measurements were then calculated by differentiate the free energy difference (Fig. 1, right):

3 $${f}_{\mathrm{AFM}}={k}_{\mathrm{B}}T\frac{\partial \mathrm{ln}\langle \mathrm{exp}(-W/{k}_{\mathrm{B}}T)\rangle }{\partial z}$$

## Simulated force-distance curves and 3D-AFM images of biopolymers

**Fig. 2:**
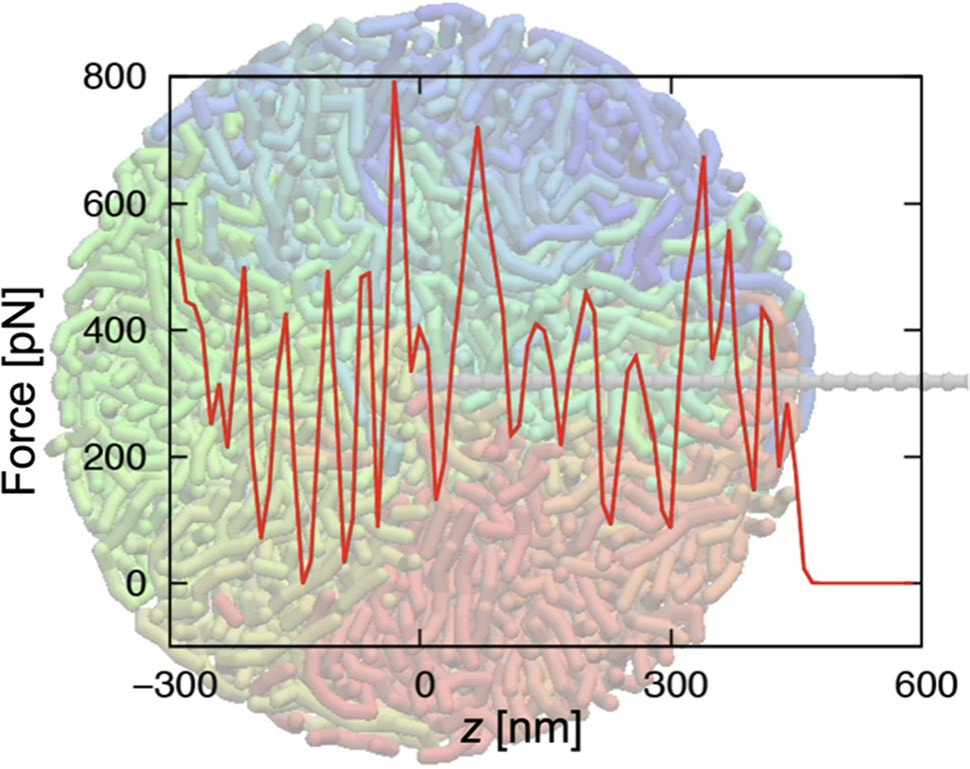
The force-distance curve inside the biopolymer and a snapshot during scanning

Using Eq. (3), force-distance curves were computed. The force-distance curve for scanning inside the biopolymer is shown in Fig. 2. This curve is calculated at the center of the globule. Because of the super coarse-graining, it takes only ~ 16 min to compute a single curve using a single core CPU (Intel Xeon Gold 2.4 GHz) when the polymer has 2000 beads and the probe has 50 beads. Several peaks are seen inside the polymer due to the large forces exerted when the probe pushes the fiber away during penetration. Thus, the peaks are not noise but actually reflect the polymer configuration in most cases (Sumikama et al. 2022).

The exceptions were as follows: first, when the polymer avoids the penetrating probe, a sufficiently detectable force does not act (Sumikama et al. 2022). It means that the fiber structure is not always resolved even though the fiber is there. This is computational evidence showing the difficulty of resolving the quickly fluctuating parts of biomolecules by AFM, which was found in the paper by Sumino et al. (2013). Another exception is broadened peaks (Sumikama et al. 2022). The probe sometimes drags the fiber down for a while, and relatively large forces continuously act during the dragging (Sumikama et al. 2022). In this case, the forces are observed where there was originally no fiber.

It is notable that almost no detectable peaks are observed when the force-distance curves are simulated using the Eq. (1) (Sumikama et al. 2022). This is because the motion of pushing away fibers is intrinsically a nonequilibrium process. The Eq. (1) is not suitable to calculate this nonequilibrium work because it assumes that the process is in equilibrium.

In order to simulate a 3D-AFM image, the force-distance curves have to be computed while changing the *x* and *y* position of the probe. This change in probe position determines the resolution of the images in the *x* and *y* directions. When scanned area is 500 nm × 500 nm and the resolution is 2.5 nm, 40,401 (= 201 × 201) force-distance curves have to be computed. Accordingly, it takes ~ 646,416 (= ~ 16 × 40,401) min (~ 450 days) to simulate a 3D-AFM image. If 100 cores are simultaneously available, simulation time is reduced to less than 5 days.

**Fig. 3:**
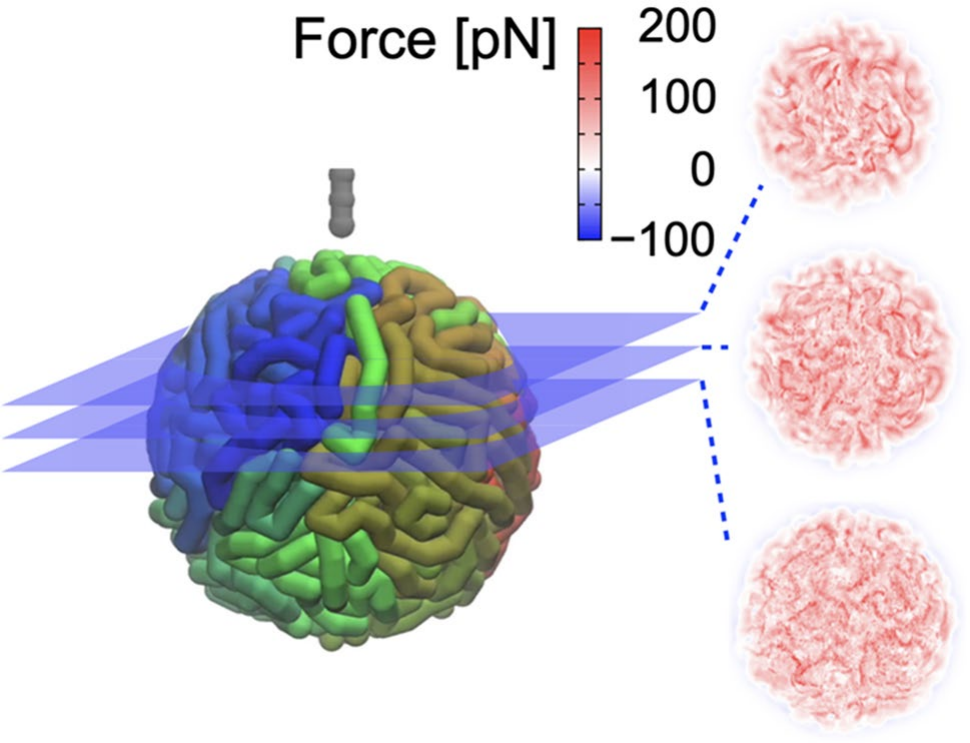
Simulated 3D-AFM image. Three *xy*-slices of the 3D-AFM image are shown with the color key for the force

Three *xy*-slices of the simulated 3D-AFM image are shown in Fig. 3. In these images, fiber structures are seen in some places where the polymer lies in the *xy*-plane and strong forces act with the penetrating probe. In a comparison of these fiber structures in the force mapping with the polymer model, it was found that polymer structures are indeed, but not completely, resolved in the 3D-AFM image (Sumikama et al. 2022). It was clarified that no fiber structure was observed when the Eq. (1) was used to simulate 3D-AFM images of the same polymer structure (Sumikama et al. 2022). This again signifies the importance of using the Jarzynski equality to simulate the forces acting in the AFM measurements of biopolymers.

The developed method was validated by comparison with 3D-AFM experiments of cytoskeleton fibers, one of the major biopolymers (Sumikama et al. 2022). 3D-AFM images of cytoskeleton fibers have been measured previously (Penedo et al. 2021). In the images, high force regions run up and down in 3D space, reflecting the fiber structure. In the *xz*-slices of the 3D-AFM images, the high force regions at the center of the fibers were extended downward because of dragging. As a result, elongated triangles were visible. This characteristic shape was reproduced in the simulated 3D-AFM images on the straight fiber structures (Sumikama et al. 2022). Therefore, the method developed was in qualitative agreement with the experiments.

Finally, the dependency of 3D-AFM images on *v*_scan_ was investigated: *v*_scan_ ranged from 0.1 to 10 µm/s, while the most probable speed (*v*_mp_) of the fiber corresponding to *v*_sample_ remained constant at 0.2 µm/s (Sumikama et al. 2022). As expected, when *v*_scan_ < *v*_mp_, the fiber can easily avoid the penetrating probe. In this case, the forces were generally too weak to be detected in real experiments. Conversely, when when *v*_scan_ ≫ *v*_mp_ (e.g., *v*_scan_ >  ~ 36*v*_mp_), strong repulsions act in many locations. This causes the static noise in the 3D-AFM images and makes it difficult to see the fiber structures in the images. Accordingly, the optimum velocity range for *v*_scan_ was found to be 10–15*v*_mp_.

## Simulated topographic images of biopolymers

As mentioned above, usual AFM images are the topographic images. In the previous paper, we developed a method to simulate topographic images by calculating the forces between the polymers and the probe (Sumikama et al. 2020). Accordingly, these images correspond to isoforce surfaces. On the other hand, once 3D-AFM images are simulated, images nearly identical to those simulated by the method developed in the paper by Sumikama et al. (2020) can be obtained within the numerical error by tracing the height at which the force equals the setpoint in the 3D-AMF images.

Simulated isoforce surface images were clearer than the equidistance surface or conventional simulated AFM images of biomolecules due to deep indentation (Sumikama et al. 2020). Unnaturally rounded shapes of biomolecules, usually seen in the equidistance surfaces, were remarkably reduced in the isoforce surface images.

One known problem in predicting AFM images of biomolecules is that the simulated size of molecules did not match to the experimental ones unless the size of the probe in simulation was reduced to 2–50% of that used in the experiments (Uchihashi et al. 2011; Rodriguez-Ramos et al. 2016; Kozai et al. 2017). As such, a theoretical background to support the validity of using such a small probe size should be clarified. The equidistance surfaces while varying the probe size were simulated and were compared to the isoforce surface. It was found that a very similar image to the isoforce surface image was obtained when the probe size was reduced to approximately 30% because the probe can approach the polymer beyond the equidistance surfaces against the repulsion between the probe and the sample due to the setpoint (Sumikama et al. 2020). This rationalizes the conventional use of a reduced probe size in predicting AFM images of biomolecules.

## Summary

In this short letter, the conventional method to compute AFM (or equidistance surface) images of biomolecules based on geometrical considerations was described. In this simulation, the positions of the biomolecules are usually fixed; thus, they never reach equilibrium and there are no nonequilibrium processes. The difficulty in computing force-distance curves for biomolecular systems using all-atom MD simulations was discussed. The method to calculate force-distance curves at the solid-water interface was also explained. The water molecules around the probe equilibrate quickly. Accordingly, the derivative of the computed free energy profiles gives the force-distance curves. Then, a recently developed method for simulating force-distance curves and 3D-AFM images of biopolymers was described. Since the scanning process of biomolecules is nonequilibrium because their motions are mostly slower than the scanning velocity, the Jarzynski equality should be employed. An optimum scanning velocity was found to exist, and at this velocity, some, but not all, polymer structures are indeed resolved in the simulated 3D-AFM images. The theoretical background of the reduced probe size in the conventional AFM simulations was clarified by comparing topographic images drawn by isoforce surfaces with the equidistance surfaces drawn by conventional AFM simulations. An important future issue is to establish a method to convert 3D-AFM images to actual biomolecular structures, which would be helpful to predict structures of biomolecules by the 3D-AFM experiments.

## Data Availability

The data are available from the corresponding author.
